# Reproducibility and FAIR principles: the case of a segment polarity network model

**DOI:** 10.3389/fcell.2023.1201673

**Published:** 2023-06-06

**Authors:** Pedro Mendes

**Affiliations:** ^1^ Center for Cell Analysis and Modeling, University of Connecticut School of Medicine, Farmington, CT, United States; ^2^ Department of Cell Biology, University of Connecticut School of Medicine, Farmington, CT, United States

**Keywords:** reproducibility, model reuse, computational modeling, ODE modeling, systems biology, SBML, segment polarity network

## Abstract

The issue of reproducibility of computational models and the related FAIR principles (findable, accessible, interoperable, and reusable) are examined in a specific test case. I analyze a computational model of the segment polarity network in *Drosophila* embryos published in 2000. Despite the high number of citations to this publication, 23 years later the model is barely accessible, and consequently not interoperable. Following the text of the original publication allowed successfully encoding the model for the open source software COPASI. Subsequently saving the model in the SBML format allowed it to be *reused* in other open source software packages. Submission of this SBML encoding of the model to the BioModels database enables its *findability* and *accessibility*. This demonstrates how the FAIR principles can be successfully enabled by using open source software, widely adopted standards, and public repositories, facilitating reproducibility and reuse of computational cell biology models that will outlive the specific software used.

## 1 Introduction

Embryonic development is characterized by frequent dynamic changes in gene expression that lead to the formation of different tissues and organs. Several patterns form during development caused by the interaction of biochemical reactions and diffusion, which was first suggested by the pioneering work of Turing (1952). Since then computational models have been used to attempt to rationalize the formation of various patterns that are crucial in development. One of these is the formation of segments in the body of insects, studied intensively in the *Drosophila* embryo (Jaeger, 2009). Insects, and other arthropods, have segmented bodies with each segment being a unit bearing a pair of appendages (such as legs). The formation of these segments during embryogenesis originates from periodic patterns of gene expression that occur in various stages. First, genes maternally expressed determine the broad regions of the body (anterior, posterior and terminal), followed by the expression of “gap genes” and then by “pair-rule genes”. Mutations on the gap genes delete contiguous segments, while mutations on pair-rule genes affect every other segment. These stages happen when cell boundaries (membranes) have not yet formed and thus multiple nuclei share a common cytoplasm (a syncytium). After separation of nuclei into separate cells, by formation of plasma membranes, the “segment polarity genes” are expressed at different levels in each cell forming a pattern that will ensure the persistent polarity of the segments throughout the rest of embryonic development.

The year 2000 is often considered to mark the beginning of the modern systems biology era. This derives from several events that happened in that year, such as the founding of the Institute for Systems Biology, the first International Conference for Systems Biology, and the publication of various articles that are now considered “classics”. One of those publications, by von Dassow et al. (2000), describes a model of the *Drosophila* segment polarity network, where a gene regulatory network operates in each one of a series of neighboring cells, with their protein products also interacting across cells (hereafter, the “SPN model”). The main conclusion, from a set of computer simulations sampling the SPN model’s parameter space, was that it is “remarkably” robust as many more random combinations of parameter values than expected give rise to the characteristic spatial gene expression pattern required for segmentation. The inference that the network structure, rather than a narrow set of parameter values, is determinant to the phenotype has been cited as a general property of systems by more than one thousand publications to this date. Another conclusion derived from those results is that the phenotype is therefore robust against perturbation of the parameters—and this has also frequently been assumed to be a general property of biological systems.

An important activity in computational systems biology is the deposition of models in public repositories using standard formats like SBML (Hucka et al., 2003) or CellML (Hedley et al., 2001). This allows any scientist to easily find and access those models and use them to run simulations or derive new ones using several compatible software applications. Through the last couple decades most classic models have been added to model repositories.

Surprisingly, being described in such a highly cited publication, the SPN model is not available in any of the four major systems biology model repositories: BioModels database (Le Novère et al., 2006; Malik-Sheriff et al., 2020), the Physiome model repository (Yu et al., 2011), JWS online (Olivier and Snoep, 2004), or the database of Virtual Cell published models (Moraru et al., 2008). To make matters worse, the software Ingeneue (Meir et al., 2002; Kim, 2009), used to create this model, is no longer available, not even through the Wayback Machine (Internet Archive, 1996). Web searches revealed an SBML implementation (Sethna, 2008) which encodes the mathematics of the model in a 4 × 6 grid of cells, but not the biochemical network.

Given the importance that the results obtained from the SPN model have had in systems biology it seems important that they be available in a well-supported software simulator and distributed in a standard format by one of the model repositories. I therefore set to encode this model with COPASI (Hoops et al., 2006; Bergmann et al., 2017) and to make sure that it was correctly implemented, use it to reproduce the simulation results of von Dassow et al. (2000), at least partially. It has been noted that reproducing results from computational studies in general (Mesirov, 2010; Peng, 2011; Stodden et al., 2016), and also computatational systems biology (Waltemath and Wolkenhauer, 2016; Mendes, 2018; Tiwari et al., 2021), is as hard as with laboratory experiments. This has also been the case here and the obstacles encountered are described below.

Through a careful examination of the publications that cite von Dassow et al. (2000), I was able to identify 15 cases where the SPN model was reused (Table 1). Only two actually reproduced their results (Ingolia, 2004; Ma et al., 2006), and another expanded the analysis to diploidy (Kim and Fernandes, 2009). Several authors used the SPN model to illustrate other issues, such as robustness (Chaves et al., 2009; Dayarian et al., 2009; Albert et al., 2011), “sloppyness” (Gutenkunst et al., 2007; Daniels et al., 2008), or new methodologies (Tegner et al., 2003; Zañudo et al., 2017; Rozum and Albert, 2018; Marazzi et al., 2022). Several software applications were used, such as the original Ingeneue (Meir et al., 2002; Kim, 2009) and Little b (Mallavarapu et al., 2009), both now unavailable, and bespoke C programs that were never distributed (Ingolia, 2004; Ma et al., 2006)—all those results are now difficult to reproduce. Only the Sethna group publications (Gutenkunst et al., 2007; Daniels et al., 2008) resulted in a version of the model that is runnable in several simulators; Marazzi et al. (2022) re-used that model and also provided a COPASI version in their GitHub repository.

**TABLE 1:** Publications that reproduced or re-used the von Dassow et al. (2000) SPN model.

References	Description	Approach	Software
von Dassow and Odell (2002)	re-used original SPN model	ODE	Ingeneue^a^
Albert and Othmer (2003)	Boolean network similar but not equal to original SPN	Boolean	unknown
Tegner et al. (2003)	Single-cell version of original SPN, without diffusive transitions	ODE	unknown
Ingolia (2004)	re-coded original SPN model	ODE	C program^b^
Ma et al. (2006)	re-coded original SPN model	ODE	C program^b^
Gutenkunst et al. (2007)	re-coded original SPN model	ODE	SloppyCell^c^
Daniels et al. (2008)	re-used code from Gutenkunst et al. (2007) ^d^	ODE	SloppyCell^c^
Chaves et al. (2009)	simplification of SPN model ODEs^e^	algebraic	N/A
Dayarian et al. (2009)	simplification of SPN model ODEs^e^	algebraic	unknown
Kim and Fernandes (2009)	re-coded diploid version of SPN model	ODE	Mathematica^b^
Mallavarapu et al. (2009)	re-coded original SPN model	ODE	Little b^a^
Albert et al. (2011)	re-coded original SPN model	algebraic	MATLAB^b^
Zañudo et al. (2017)	re-used original SPN model	ODE	Python^b^
Rozum and Albert (2018)	re-coded single-cell version of original SPN model	algebraic	Python
Marazzi et al. (2022)	re-used SBML model from Daniels et al. (2008) ^d^	ODE	COPASI

^a^ Software no longer available.

^b^ Code not publicly available.

^c^ Software available from https://sloppycell.sourceforge.net/.

^d^ SBML version available from https://sethna.lassp.cornell.edu/Sloppy/vonDassow/model.html.

^e^ Used a square grid of cells.

This exercise identifies issues that hinder reproducibility and reuse of biomodels, and illustrates how they can be overcome with modern open science practices addressing the FAIR principles (Wilkinson et al., 2016). Reproducing it required a certain level of “archeological” craft to find missing parts. I hope that this also serves as a demonstration of procedures that make models usable beyond the lifetime of the software that created them. Of course, the SPN model was an important and early application of computational systems biology to developmental biology, and reproducing its results is also not irrelevant.

## 2 Methods

### 2.1 Software

**FIGURE 1:**
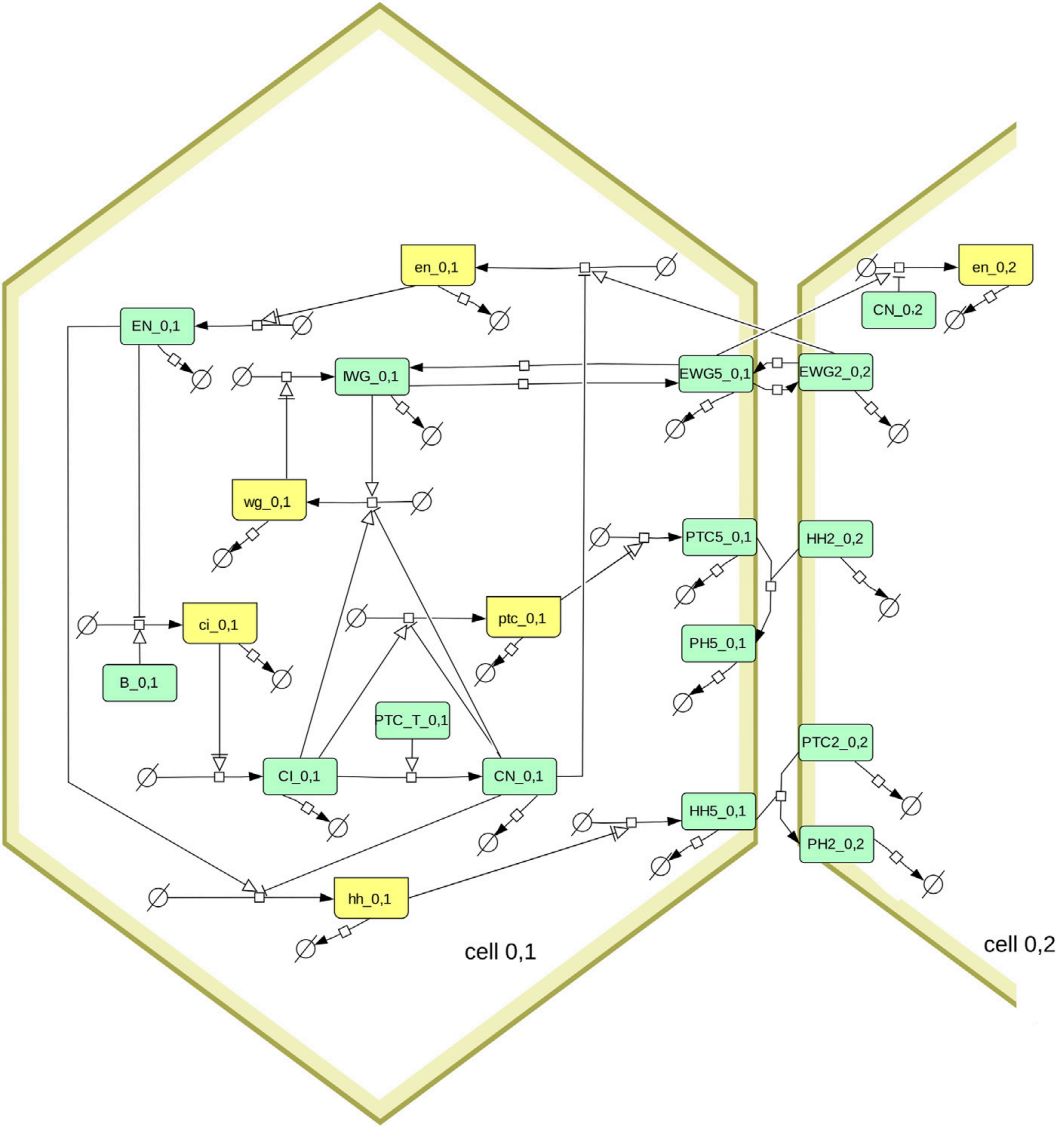
Diagram of the segment polarity network following the SBGN standard (Le Novère et al., 2009; Touré et al., 2021). Boxes in light green represent proteins, boxes in yellow represent mRNA. The full model includes several hexagonal cells, this diagram shows only one (cell_0,1) and its interactions with one of its neighbors (cell_0,2). Note that the membrane proteins (EWG, PTC, HH, and PH) exist in six pools, one for each side of the hexagonal cell. Only the proteins in side 5 are shown on the diagram, as well as the proteins on side 2 of the neighboring cell. The membrane proteins are allowed to diffuse between sides of the hexagon, which is also not shown here (*eg.* EGW5_0,1 can transfer reversibly to EGW4_0,1 and EGW6_0,1). The box labeled PTC_T_0,1 represents the sum of all PTC species (from the six sides of the membrane of cell_0,1).

Model simulations and parameter sampling were carried out with COPASI version 4.39 (Hoops et al., 2006; Bergmann et al., 2017, RRID:SCR_014260), Virtual Cell version 7.5.0 (Schaff et al., 1997; Moraru et al., 2008, RRID:SCR_007421), Tellurium version 2.2.7 (Choi et al., 2018) that uses libRoadRunner version 2.3.2 (Welsh et al., 2023, RRID:SCR_014763), and AMICI version 0.11.25 (Fröhlich et al., 2021), which was accessed through runBioSimulations (Shaikh et al., 2021, RRID:SCR_019110). The model file was constructed with python scripts using the BasiCO package that interfaces with COPASI (Bergmann, 2023). Simulations were run at the local high-performance computing cluster using the Cloud-COPASI web interface (Kent et al., 2012). Results were visualized with COPASI, with Gnuplot version 5.4.3 (Williams and Kelley, 2022, RRID:SCR_008619), or with the Python libraries Seaborn (Waskom, 2021) (RRID:SCR_018132) and Matplotlib (Hunter, 2007, RRID:SCR_008624). The SBGN diagram of Figure 1 was created using Cell Designer version 4.4 (Funahashi et al., 2003, RRID:SCR_007263) and then edited with Inkscape version 1.1 (RRID:SCR_014479).

### 2.2 Model

The model used here is the segment polarity network model described by von Dassow et al. (2000). Briefly it represents a hexagonal array of cells, where each cell can express various genes (*wingless*, *engrailed*, *hedgehog*, *cubitus interruptus*, and *patched*) and where their protein products interact within a cell, and across neighboring cells. Figure 1 depicts the interaction network using the SBGN standard (Le Novère et al., 2009; Touré et al., 2021). Note that von Dassow et al. (2000) analyze two versions of this model, one having less interactions than the other. Here we only look at their full model (i.e., including the dashed arrows in the diagram of their Box 1). Since a 1 × 4 grid of cells is enough to replicate the results (von Dassow et al., 2000), that was used here to obtain all results.

My implementation of the model was first created for the widely used software COPASI (Hoops et al., 2006; Bergmann et al., 2017) through a Python script that creates a model with arbitrary number of cells at the user’s desire. A second script was created to generate the same model with only one cell, where the interacting species from neighboring cells are included as fixed concentrations. COPASI generates the full set of ODEs automatically based on the network and reaction kinetic rate laws. Unlike the SBML version from Sethna (2008), here we have the full reaction network, not just the differential equations. A small formal difference between this version and the original SPN model, is that COPASI expresses ODEs in terms of the species amounts rather than concentrations, but since the cell volumes are not variable this makes no difference and both sets of equations are equivalent.

The model makes extensive use of Hill-type functions where various terms appear in the form *base* ^*exponent*^. This is often problematic in IEEE floating point since, for non-integer exponents, those operations are carried out based on the equivalence:

$${\text{base}}^{\text{exponent}}=e^{\text{exponent}\times \log\left(\text{base}\right)}.$$ (1)

Therefore, calculations fail when *base* is negative, even if infinitesimally small (generates a NaN, which in COPASI is translated to an error “Invalid state”). Unfortunately, due to the nature of predictor-corrector integration algorithms, this can easily happen during a time course integration if one species concentration becomes very close to zero. In order to avoid this problem one can use a kind of “guarded” exponentiation:

$${\text{base}}^{\text{exponent}}≃\max{\left(\epsilon ,\text{base}\right)}^{\text{exponent}},\epsilon >0.$$ (2)

Applying this protection to the model changes the rate laws. For example, the rate law for transcription with inducer-repressor pair changes from the original:

$$V\cdot \frac{I\cdot {\left(1-\frac{R^{h_{2}}}{k_{2}^{h_{2}}+R^{h_{2}}}\right)}^{h_{1}}}{k_{1}^{h_{1}}+I\cdot {\left(1-\frac{R^{h_{2}}}{k_{2}^{h_{2}}+R^{h_{2}}}\right)}^{h_{1}}}$$ (3)

to the alternative:

$$V\cdot \frac{I\cdot \max{\left(\epsilon ,1-\frac{\max{\left(\epsilon ,R\right)}^{h_{2}}}{k_{2}^{h_{2}}+\max{\left(\epsilon ,R\right)}^{h_{2}}}\right)}^{h_{1}}}{k_{1}^{h_{1}}+I\cdot \max{\left(\epsilon ,1-\frac{\max{\left(\epsilon ,R\right)}^{h_{2}}}{k_{2}^{h_{2}}+\max{\left(\epsilon ,R\right)}^{h_{2}}}\right)}^{h_{1}}}.$$ (4)

The terms $k_{1}^{h_{1}}$ and $k_{2}^{h_{2}}$ are not protected by a “guard” because *k*_1_ and *k*_2_ are constants that are always positive. In the results presented here I have used *ϵ* = 10^–80^, which reduced the incidence of simulations with NaNs from around 10%–0.1%. von Dassow et al. (2000) did not describe how they avoided this problem within the software Ingeneue. Use of these alternative rate laws was necessary for the random parameter sampling, but for specific time course simulations one can almost always use the original rate laws as described in von Dassow et al. (2000).

Several aspects of the original SPN model were not fully described by von Dassow et al. (2000) and I have had to resort to later publications to infer what they could be. For the sake of complete transparency, here are all the details that had to be inferred from sources other than the original article.

• Parameter *H*_*EWG*_ does not feature in the differential equations of the Supplementary Material S1 or in von Dassow and Odell (2002), instead there the proteins *EWG* and *IWG* have the same half-life (*H*_*IWG*_). However the parameter is clearly described as one of the 48 parameters sampled in Meir et al. (2002), from the same group. Thus in my implementation *EWG* has its own half-life *H*_*EWG*_.
• The identity of the 48 parameters that are sampled was not described unequivocally. There are in fact 53 parameters in the model (when considering 4 cells), so while 46 were obvious from their Supplementary Table S1, the other 2 could have been any of the remaining 7 … Again, a Figure in Meir et al. (2002) provided the identity of the 48 parameters (which include the one mentioned in the previous bullet).
• The ranges for parameter samplings are provided in Supplementary Table S1, however it missed including the ranges for parameters *PTC*_0_ and *HH*_0_. Kim (2009) mentions this range as 1–1000 (their table 3, parameters “max”), while an Ingeneue network file (named spg1_01_4cell.net), recovered from the Internet Archive (Kim, 2010), suggests it could be 10^3^–10^6^. I ran simulations with both ranges, and the range 1–1000 produces results closer to those reported by von Dassow et al. (2000).
• The score function used to identify parameter sets that result in the desired properties was described without sufficient detail. This scoring function is a composite of a function to identify the gene expression pattern (Eq. 15 of their Supplementary Material S1), and another to detect stable stripes (Eq. 16 of their Supplementary Material S1); the final score being the largest of these two. The text does not specify clearly what the symbols of Eq. (16) mean, particularly the *StripeScore*. Thus I only used Eq. (15) for scoring. By definition my results should identify more parameter sets than the full scoring criterion (since we are looking for scores below a threshold of 0.2).
• The initial conditions probed in each line of Table 1 of the original paper are not specified exactly, instead they provide ranges, such as <20 % value, or 20%–60%, not saying whether the values used were random within that range or some actual specific values. I used 0.15 for when they indicate <20 %, 0.4 for when they specify 20%–60%, and 0.9 when they specify 60%–100%. For the “degraded” initial condition this is even more problematic as they only provided a bar chart without axes, rather than actual values. The values I used here are specified in the Python code and in the COPASI and SBML files for the time course described below.

As described in the Interoperability section of Results, below, the model can be exported from COPASI in standard formats, particularly the systems biology markup language (SBML, Hucka et al., 2003; Keating et al., 2020) and the OMEX format (Bergmann et al., 2014) containing a SBML file for the model and a SED-ML (Waltemath et al., 2011b) file with the simulation specification.

## 3 Results

### 3.1 Reproducibility

It is rather unfortunate that the term “reproducibility” has itself been used with various different meanings. This confusion in terminology was discussed in detail by Goodman et al. (2016), Plesser (2018), Miłkowski et al. (2018), and especially Barba (2018). As previously (Mendes, 2018), I will follow the definitions of Goodman et al. (2016), which specifies three distinct types of reproducibility.

• *reproducibility of methods* requires one to be able to exactly reproduce the results using the same methods on the same data;
• *reproducibility of results* requires one to obtain similar results in an independent study applying similar procedures;
• *reproducibility of inferences* requires the same conclusions to be reached in an independent replication potentially following a different methodology.

Because the software Ingeneue, originally used to build and simulate the SPN model, has now disappeared from circulation, reproducibility of methods can no longer be effectively carried out. In a later publication von Dassow and Odell (2002) appear to have reproduced the results with the same software (see Table 1), however since these are the original authors, that can hardly be seen as independent verification. Of all the works listed in Table 1, only Ingolia (2004) and Ma et al. (2006) can be seen as independent reproductions of the original results. Unfortunately those two publications used their own C programs but did not publish them. It was work in Sethna’s lab (Gutenkunst et al., 2007; Daniels et al., 2008) that resulted in an electronic version of the model being created in the SBML format that is still available (see notes to Table 1), and which was re-used by Marazzi et al. (2022). However this SBML implementation coded the ODEs directly without representing the reaction network, an important limitation.

**FIGURE 2:**
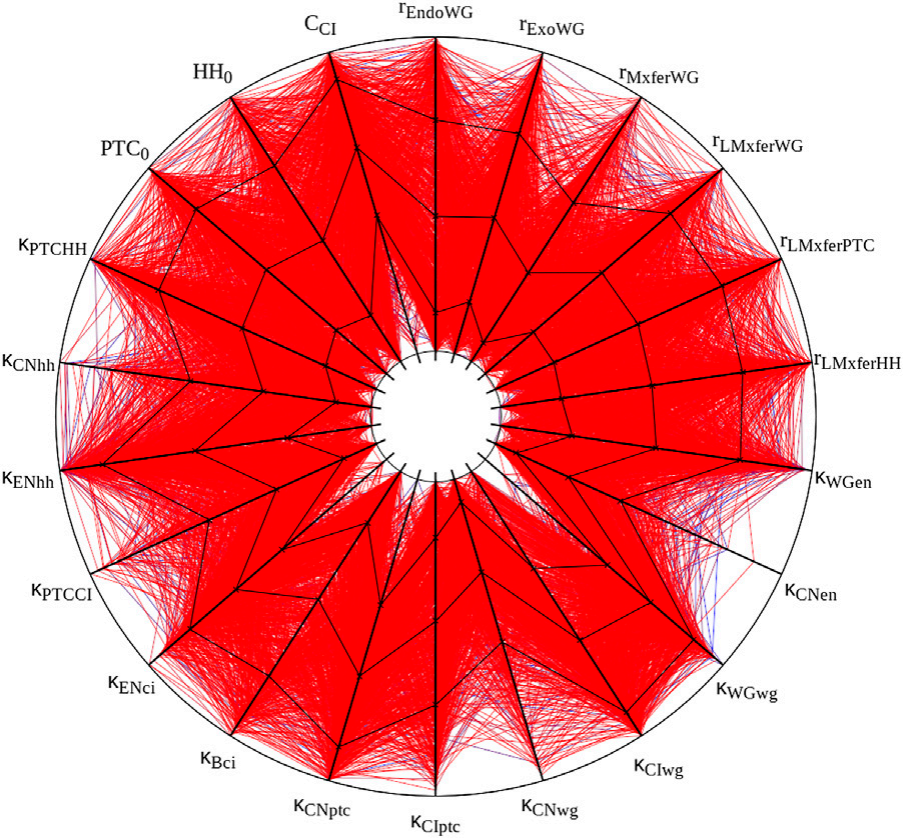
Graphic representation of ”solutions” obtained with crisp initial conditions. All 1,015 parameter sets with a score below 0.2 are displayed. Black lines plot mean and standard deviation. Each spoke represents the log-scale range of one parameter. Half-lives and cooperativity coefficients are omitted, as in Panel 2A of von Dassow et al. (2000). This figure was created with the open source software Gnuplot and its source is included with the available data sets (see Data Availability Statement).

I attempted to reproduce the results of Table 1 in von Dassow et al. (2000), displayed in our Table 2. Overall these results match the original ones fairly well. There are some discrepancies in two samplings, but these are likely due to the uncertainty on the actual initial values, as pointed out in Methods. Bear in mind that these are very small samples of a 48-dimensional parameter space and the differences may just be due to random sampling. Figure 2 displays the succesful parameter sets in the sampling with crisp initial conditions, corresponding to Figure 2A in von Dassow et al. (2000). Careful comparison between the Figure and the original one reveals similar distributions. For example, in both cases *κ*_*CNen*_ rarely takes large values. The conclusions taken by von Dassow et al. (2000) would not change if their Figure 2A was substituted by this Figure 2. Taking these results together, I propose that the current implementation of the SPN model matches the results of the original—*reproducibility of results.*

**TABLE 2:** Frequency of solutions as a function of initial conditions.

	Von Dassow et al. (2000)			This work		
Initial conditions	Hits	Tries	Hit rate	Hits	Tries	Hit rate
Crisp	1,192	240,000	1/201	1,015	239,272	1/236
Degraded	149	750,000	1/5,000	22	749,988	1/34,090
Crisp, plus ubiquitous low-level *ci* and *ptc*	110	41,258	1/375	91	41,941	1/461
3-cell band of *ci*, *wg* stripe on posterior margin	69	40,338	1/585	97	41,994	1/433
3-cell band of *ptc*, *en* stripe on anterior margin	127	36,196	1/285	102	37,994	1/372
3-cell band of *ptc*, out-of-phase 3-cell band of *ci*	16	226,084	1/14,130	168	229,996	1/1,369
10.5281/zenodo.7772570 Close to target pattern	464	21,526	1/46	556	21,992	1/39

### 3.2 Interoperability

To demonstrate that this implementation of the SPN model is interoperable across different software, a specific time course was chosen to be run by several simulators (herafter named *timecourse1*). One of the successful parameter sets generated in the random sampling with the “degraded” initial condition was chosen and saved as a native COPASI file, an SBML Level 3 Version 1 file (Hucka et al., 2018), and an OMEX file (Bergmann et al., 2014). Both the COPASI and OMEX files include the specification of the time course (end time of 1100 time units, sampled every 5 time units), though the SBML file requires that time course to be specified separately in the destination simulator.

Timecourse1 was simulated in four different software tools: COPASI, Virtual Cell (Schaff et al., 1997; Moraru et al., 2008), Tellurium (Choi et al., 2018), and AMICI (Fröhlich et al., 2021). It was run locally with COPASI, Virtual Cell, and Tellurium, and through the web service runBioSimulations (Shaikh et al., 2021) with AMICI. COPASI used the native file format, Tellurium used the SBML (through a small Python script runTellurium.py), while Virtual Cell and AMICI used the OMEX file.

**FIGURE 3:**
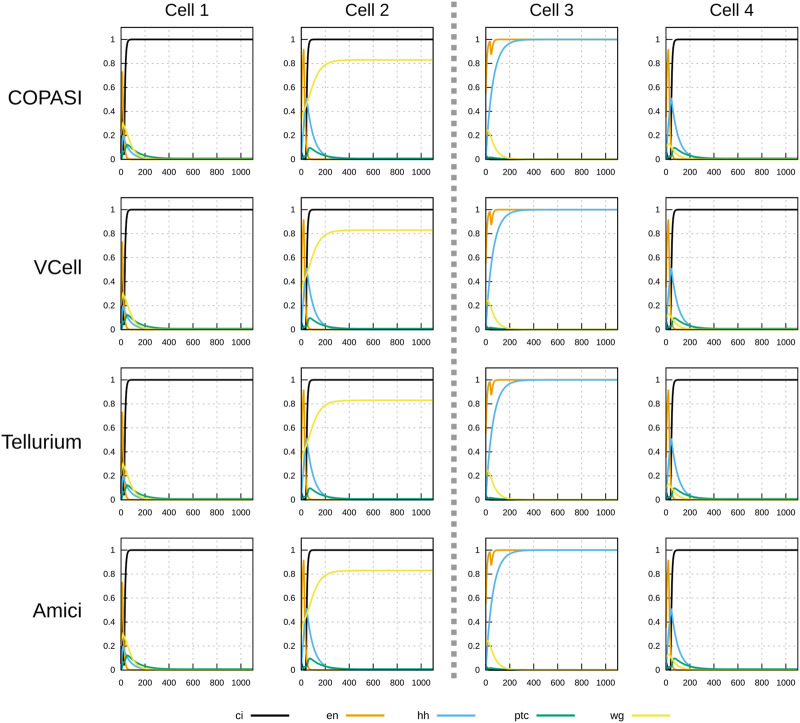
Time course simulation of mRNA species in a 1×4 arrangement of cells using a parameter set obtained by random sampling from the “degraded” initial condition (see Table 2). Columns represent the different cells; the middle dashed line separating cell 2 and cell 3 represents a parasegmental boundary. Displayed in each plot are the time evolution of all mRNA species in that cell. Note the formation of the expected segment polarity pattern around the parasegmental boundary, with high levels of *wingless* and *patched* in cell 2, and high levels of *engrailed* and *hedgehog* in cell 3. Each row corresponds to simulations carried out by different software. COPASI used the LSODA algorithm with absolute tolerance 10^–13^ and relative tolerance 10^–8^. Virtual Cell used a fixed step size Adams-Moulton algorithm (step size 0.1). Tellurium used CVODE non-stiff algorithm (variable step size, variable order Adams-Moulton) with absolute tolerance of 10^–12^ and relative tolerance of 10^–6^. AMICI used CVODES with absolute tolerance of 10^–16^ and relative tolerance of 10^–8^. Results from the four simulators are visibly the same.

**FIGURE 4:**
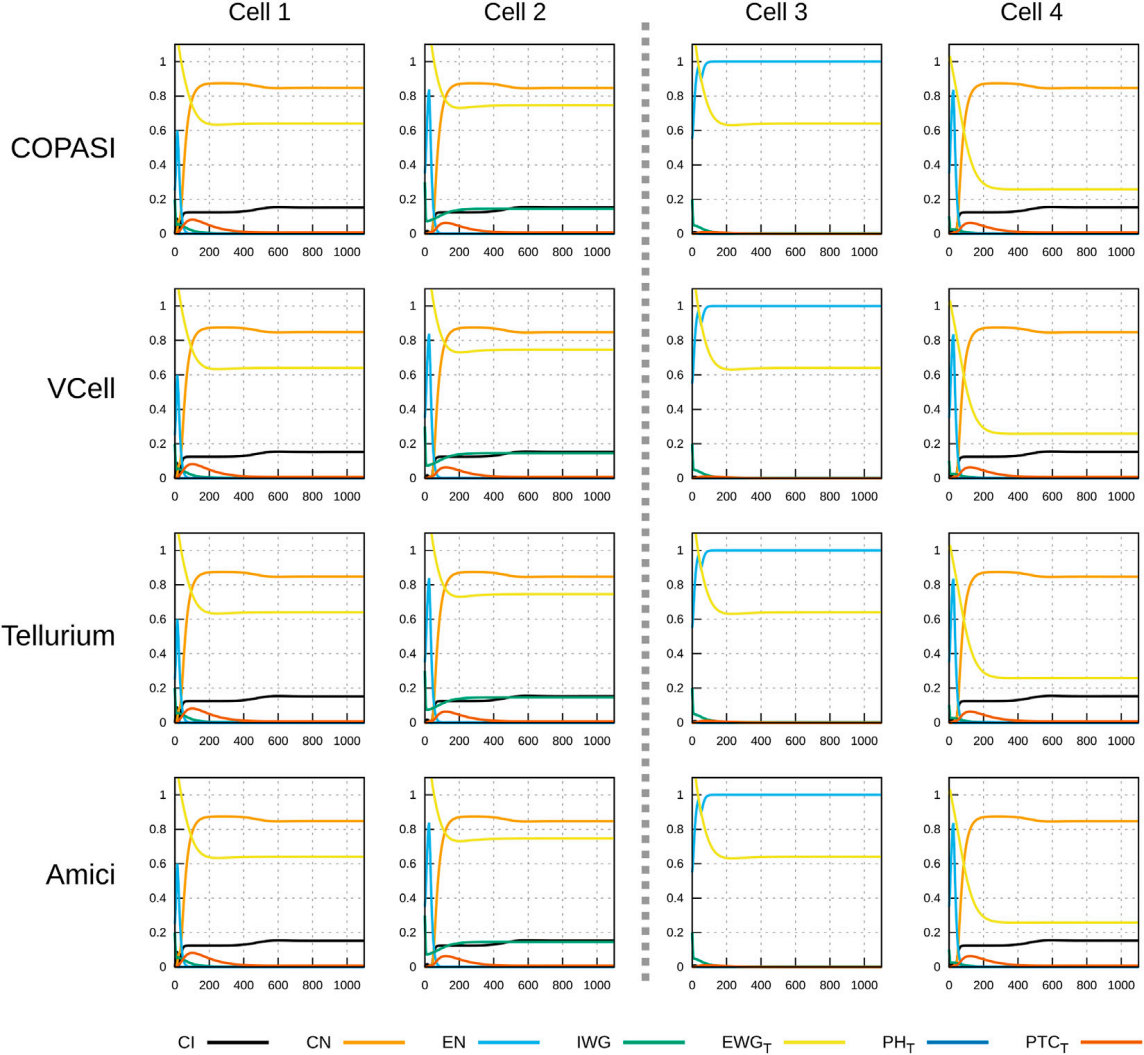
Time course simulation of protein species as in Figure 3. Displayed in each plot are the time evolution of some of the protein species in that cell. Species *EWG*_*T*_ represents the total amount of *EWG* protein (product of *wingless*) located in the membranes of the six neighboring cells to the one displayed; *PH*_*T*_ is the sum of all patched–hedgehog complexes located in the six sides of that cell’s membrane, and *PTC*_*T*_ is the sum of all free patched receptor located in the six sides of that cell’s membrane. Each row corresponds to simulations carried out by different software with different algorithms. As in Figure 3, there are no visible differences in the results of the four simulators.

Figures 3, 4 display the time course simulations obtained with four different software. There are no visible differences in the trajectories displayed confirming that these packages are all equally able to reproduce the results. Note that different ODE solvers were used by each one: COPASI used LSODA (Petzold, 1983), Virtual Cell used a fixed-step size Adams-Moulton method (Han and Han, 2002), Tellurium used CVODE (using the Adams-Moulton variable order, variable step size method) and AMICI used CVODES, both part of the SUNDIALS suite (Hindmarsh et al., 2005).

### 3.3 Findability and accessability

To promote findability and accessibility, the model files and associated scripts are made available through the following channels: a) a GitHub repository https://github.com/pmendes/models/tree/main/vonDassow2000, b) a Zenodo accession DOI (doi:10.5281/zenodo.7772570), c) a submission to the Biomodels database (MODEL2304060001), and d) model files deposited in the database of public Virtual Cell models. Note that the complete result files are only accessible through Zenodo since several files were larger than the limit at GitHub.

### 3.4 Reuse

To demonstrate how the model can be reused for different purposes, I decided to ask the question “how often do parameter sets of the SPN model have multiple steady states?” Earlier von Dassow and Odell (2002) and especially Ingolia (2004) proposed that the robustness of pattern formation in the SPN model is due to multi-stability of steady states. Ingolia (2004) showed this in SPN models of a single cell (where the interacting species from the neighboring cells are kept constant). Here I investigate the answer to this question in a 1 × 4 array of cells. The strategy I used is as follows.

1. Generate *p* random sets of parameter values;
2. For each set of parameter values generate *i* random sets of initial conditions and calculate their steady state by integration;
3. Determine how many sets of parameter values produced more than one steady state.

COPASI can easily to carry out such a study directly with the *Parameter scan* and *Steady state* tasks. The steady state task was applied here disabling the Newton method and therefore only using ODE integration to find the steady state reachable from the initial conditions (the steady state resolution was set to 10^–4^ and the criterion used was “distance and time”). With the parameter scan task, 5,000 random parameter sets were sampled, using the same rules as in Section 3.1 above. Then, for each parameter set, it sampled 15 random initial conditions. Since we use a model of 1 × 4 array of cells, the initial conditions are composed of 132 species concentrations that were sampled in the interval [0,1].

From the 5,000 random parameter sets generated, 3,387 had at least one steady state (the remainder are likely to contain limit cycles, but this was not investigated). Of those 3,387 parameter sets with steady states, 498 contained more than one steady state. This rate of 1/10 parameter sets displaying multistability is not entirely surprising given the study by Ingolia (2004) which highlighted the positive feedbacks contained in the SPN model. Nevertheless it is interesting to investigate if these 498 parameter sets have special characteristics *versus* the other 2,889 that have only one steady state.

**FIGURE 5:**
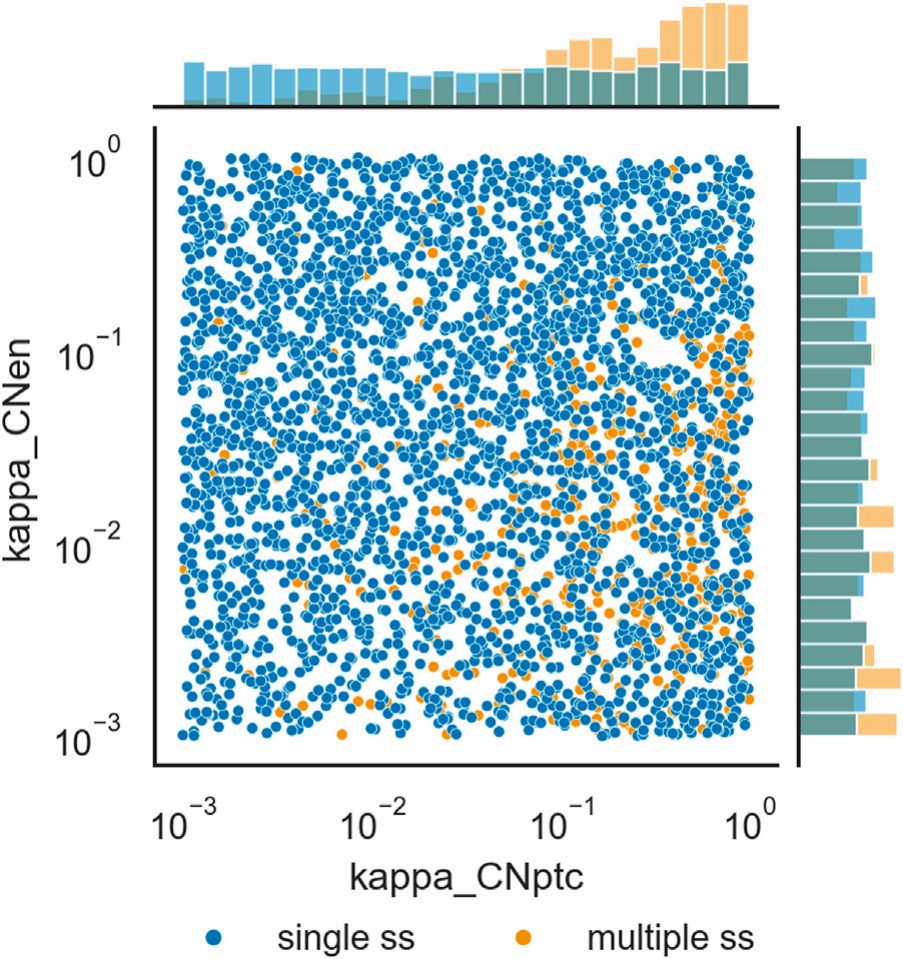
Distribution of values of *κ*_*CNptc*_ and *κ*_*CNen*_ originating single steady states, *versus* those originating multiple steady states. Scatterplot of values of the two parameters and histograms of their distribution. Darker blue circles represent parameter sets for which only one steady state was identified, lighter orange circles represent parameter sets for which more than one steady state could be identified.

The distributions of parameter values that support multiple steady states was compared with those that appear to only support a single steady state. Calculation of the relative change in the median values for each parameter in the single steady state set *versus* the multiple steady state set revealed that only *κ*_*CNptc*_ shows a large difference, with a median 5-fold larger in the multiple steady state set than in the single steady state set. Three others have much lower differences: *κ*_*CNen*_ 0.7-fold smaller, *κ*_*CIptc*_ 0.46-fold smaller, and *HH*_0_ 0.45-fold smaller. The other 44 parameters have smaller differences. Figure 5 depicts the distributions of values of *κ*_*CNptc*_ and *κ*_*CNen*_ for the two data sets. Supplementary Figures S1–S3 depict histograms for all of the 48 parameters. There seems to be very few parameter sets that lead to multiple steady states with low values of *κ*_*CNptc*_, while many more have high values for this parameter. This suggests that in order to achieve multiple stability the repression of *patched* (*ptc*) transcription by the truncated protein product of *cubitus interruptus* (*CN*) should be weak. Note that there is another negative feedback loop between these two genes, through induction of *ptc* transcription by the full length *cubitus interruptus* protein (*CI*).

## 4 Discussion

It is widely recognized that there is a “reproducibility crisis” in science (Baker, 2016) that includes computational science (Mesirov, 2010; Peng, 2011; Stodden et al., 2016) and indeed computational modeling of biological systems (Waltemath and Wolkenhauer, 2016; Mendes, 2018; Tiwari et al., 2021). I and others argue that reproducibility of results obtained from computer simulations of biological models (biomodels) could be enhanced by using open source software (Ince et al., 2012; Mendes, 2018) that implement widely adopted standards (Waltemath and Wolkenhauer, 2016; Blinov et al., 2021; Porubsky et al., 2021), which are part of various sets of rules proposed in the last 2 decades (Le Novère et al., 2005; Waltemath et al., 2011a; Lewis et al., 2016; Porubsky et al., 2020). Adoption of such practices, though, will only become widespread when enforced by publishers (Schnell, 2018; Stodden et al., 2018) and funding agencies (Yale Law School Roundtable Participants, 2010). A recent move by the US National Institutes of Health to enforce standards for data management (National Institutes of Health, 2020) is an encouraging move in that direction.

While reproducibility is a fundamental part of the scientific process (Popper, 1959), another important aspect is that new discoveries are almost always dependent on previous results, methodologies, and theories. To facilitate reuse of scientific data the community is increasingly adopting the so-called FAIR data principles (Wilkinson et al., 2016) which promote *Findability*, *Accessibility*, *Interoperability*, and *Reuse* of data. While biomodels are usually seen as mathematics or software, they are operationally complex data objects and these principles ought to apply to them as well. Here I reproduced the reaction network, ODE model and associated simulations described in the classic systems biology paper by von Dassow et al. (2000) with the software COPASI. I then exported the model and simulation specifications in community-derived standard formats that are supported by many software applications. Finally these files were contributed to model and data repositories. This essentially makes the model available to be manipulated by a large number of software applications, not only extant but likely future ones. Even if the standards used here will be abandoned in the future, it is most likely that converters would be developed to upgrade models to the new standards. Model and data repositories are also expected to last a long time. Thus this classic systems biology and development model is now available to a wide community, enabling its re-use for many decades.

As in previous case studies (e.g., Jablonsky et al., 2011; Tiwari et al., 2021), not all required information to reproduce the model and simulations were available in the original publication. Fortunately, there were subsequent publications by the authors and other members of their teams that hinted at the missing pieces. In some cases there is still uncertainty whether I made the correct choices, however the results obtained (Figure 2) are sufficiently close to the original that these choices are at least validated to be highly plausible. This supports previous suggestions (Claerbout and Karrenbach, 1992; Hothorn and Leisch, 2011; Stodden et al., 2016) that true computational reproducibility requires availability of electronic executable versions. Unfortunately textual descriptions are almost always deficient in details, as it is only too easy to miss something.

While the missing information in von Dassow et al. (2000) could be seen as a negative, I note that at the time the software Ingeneue was distributed together with files that allowed reproduction of the results. Additionally the model was actually described in great detail, so much that I was able to re-implement it. It is not uncommon to come across cases where even the model equations are not listed (see, e.g., Hübner et al., 2011, for a survey). However, this also highlights that publishing an electronic version alone is not guarantee that others in the future will be able to use it. In this case the software Ingeneue is no longer distributed and thus the electronic version is essentially lost (I could have tried to seek a copy from the original authors but I decided not to do so in order to test whether I could reproduce it with the information available). Publication of models in a widely used standard format is essential, as only this will assure the model to be interoperable by future software. Again, this is not a criticism of this 23 year-old publication, since at that time the relevant standards were nonexistent.

In conclusion: we have all the tools needed to make computational systems biology models FAIR. They should be encoded in standard formats with relevant metadata and deposited in widely used repositories. Only this will assure that future researchers will be able to study and re-use these models. Any other option, such as only describing model equations, making the model available “upon request”, or non-standard electronic encodings of the model will likely be lost within a decade or less.

## Data Availability

The datasets presented in this study can be found in online repositories. The names of the repository/repositories and accession number(s) can be found below: GitHub: https://github.com/pmendes/models/tree/main/vonDassow2000 Zenodo: https://zenodo.org/record/7772570 Biomodels: https://www.ebi.ac.uk/biomodels/MODEL2304060001.
