# Supplementary material for: Reproducibility and FAIR principles: the case of a segment polarity network model
Source: Front Cell Dev Biol. 2023 Jun 6;11:1201673. doi: 10.3389/fcell.2023.1201673 (PMC10279958; doi:10.3389/fcell.2023.1201673)
Supplement: Supplementary file 1 [file DataSheet1.PDF]

# Supplemental Figures for "Reproducibility and FAIR Principles: The Case of a Segment Polarity Network Model"

Pedro Mendes

April 2023

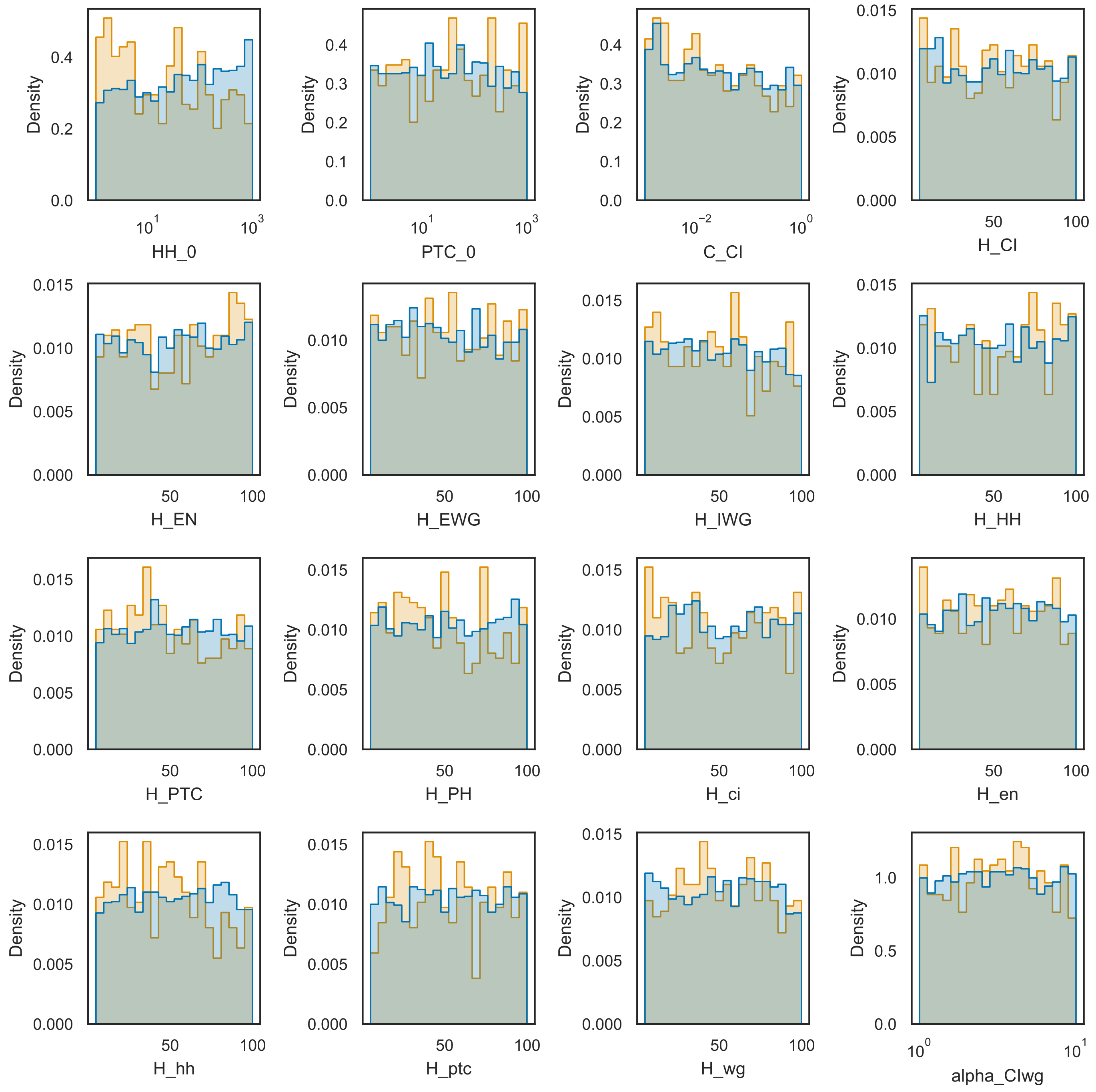


Figure S1: Distributions of paramater values that result in a single steady state (blue) and multiple steady states (orange).

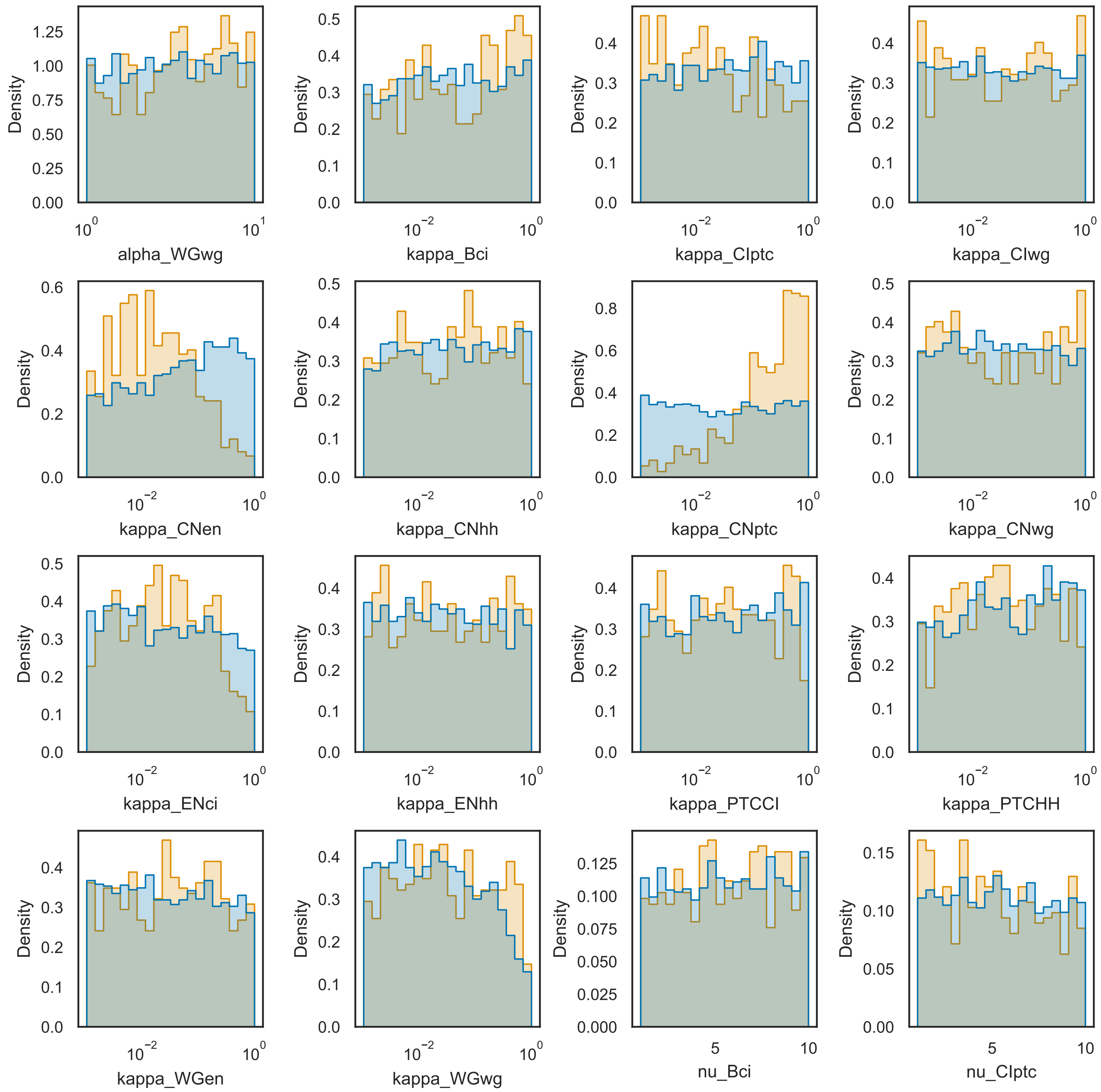


Figure S2: Distributions of paramater values that result in a single steady state (blue) and multiple steady states (orange).

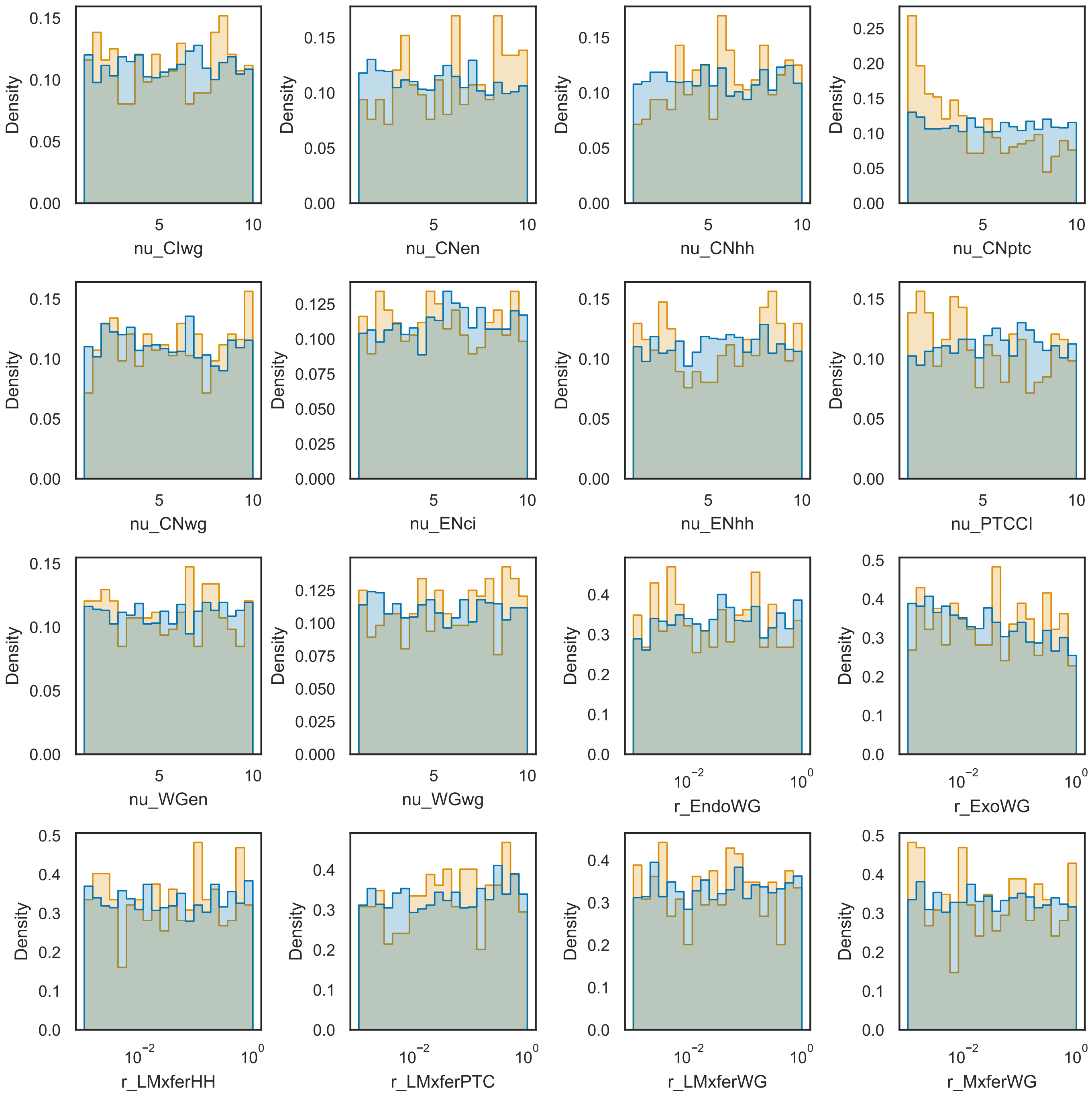


Figure S3: Distributions of paramater values that result in a single steady state (blue) and multiple steady states (orange).
